# The Effect of Unenhanced MRI on the Surgeons’ Decision-Making Process in Females with Suspected Appendicitis

**DOI:** 10.1007/s00268-016-3626-7

**Published:** 2016-08-05

**Authors:** C. M. P. Ziedses des Plantes, M. J. F. van Veen, J. van der Palen, J. M. Klaase, H. A. J. Gielkens, R. H. Geelkerken

**Affiliations:** 1 Department of Radiology, Medical Spectrum Twente, PO Box 50000, 7500 KA Enschede, The Netherlands; 2 Department of Surgery, Medical Spectrum Twente, PO Box 50000, 7500 KA Enschede, The Netherlands; 3 Department of Epidemiology, Medical Spectrum Twente, PO Box 50000, 7500 KA Enschede, The Netherlands; 4 Faculty of Science and Technology, Experimental Centre of Technical Medicine, University of Twente, PO Box 217, 7500 AE Enschede, The Netherlands; 5 Department of Radiology, SHO Center for Medical Diagnostics, 6883 JP Velp, The Netherlands; 6 Department of Surgery, UMCG, PO Box 30001, 9700 RB Groningen, The Netherlands

## Abstract

**Background:**

This prospective study evaluated the impact of the results of unenhanced magnetic resonance imaging (MRI) on the surgeon’s diagnosis of acute appendicitis in potentially fertile females.

**Methods:**

112 female patients, aged 12–55, with suspected appendicitis underwent MRI of the abdomen. At three defined intervals; admission and clinical re-evaluation before and after revealing the MRI results, the surgeon recorded the attendance of each patient in operative treatment, observation or discharge. Appendicitis was confirmed or declined by pathology or by telephone follow-up in case of non-intervention.

**Findings:**

Appendicitis was confirmed in 29 of 112 patients. At admission the surgeon’s disposition had a sensitivity of 97 % and specificity of 29 %. After knowing the MRI results, sensitivity was 97 % and specificity 64 %. The sensitivity and specificity of MRI alone were 89 and 100 %, with a negative and positive predictive value of 96 and 100 %, respectively.

**Conclusion:**

We believe that MRI should perhaps be standard in all female patients during their reproductive years with suspected appendicitis. It avoids an operation in 32 % of cases and allows earlier planning for patients with an equivocal clinical picture. Trial number: OND1292733 (Narcis.nl).

## Introduction

Acute appendicitis is the most common indicator for emergency abdominal surgery. Early appendicitis may present itself atypically and it is difficult to distinguish from a myriad of gastrointestinal, genitourinary and gynaecological conditions [1]. A healthy appendix is found in 15 % of patients clinically suspected for appendicitis, rising to 45 % in women during their reproductive years [2, 3]. Delays in diagnosis increases the risk of appendiceal perforation, which increases the danger of postoperative complications up to 39 %, as compared to 8 % for non-perforated appendicitis [4, 5]. Therefore, a timely and proper diagnosis of appendicitis remains urgent and challenging, even for experienced clinicians.

Advances in radiology, like ultrasonography (US), computed tomography (CT) and magnetic resonance imaging (MRI) can help the clinician to quickly determine the correct diagnosis in patients with suspected appendicitis. The applicability of US in diagnosing appendicitis is good but the accuracy of US is operator dependent [6, 7]. The precision of a CT scan is adequate, however the ionising radiation is a disadvantage, especially in younger patients [8]. The present study investigates the clinical value of unenhanced MRI in females during their reproductive years with clinically suspected appendicitis.

## Patients and methods

In an 18-month period, all female patients receiving surgical consultation for possible acute appendicitis in a large regional teaching hospital were evaluated for inclusion into this prospective cohort study. The inclusion criteria were a clinical suspicion of appendicitis and female sex in the age of 12 through to 55 years that were presented at the emergency department. Patients were excluded if informed consent was not obtained, if the patients were pregnant or in case of a known contraindication for MRI. The exclusion of patients that met the inclusion criteria was registered. The local institutional ethics committee approved this study.

### Study design

**Fig. 1:**
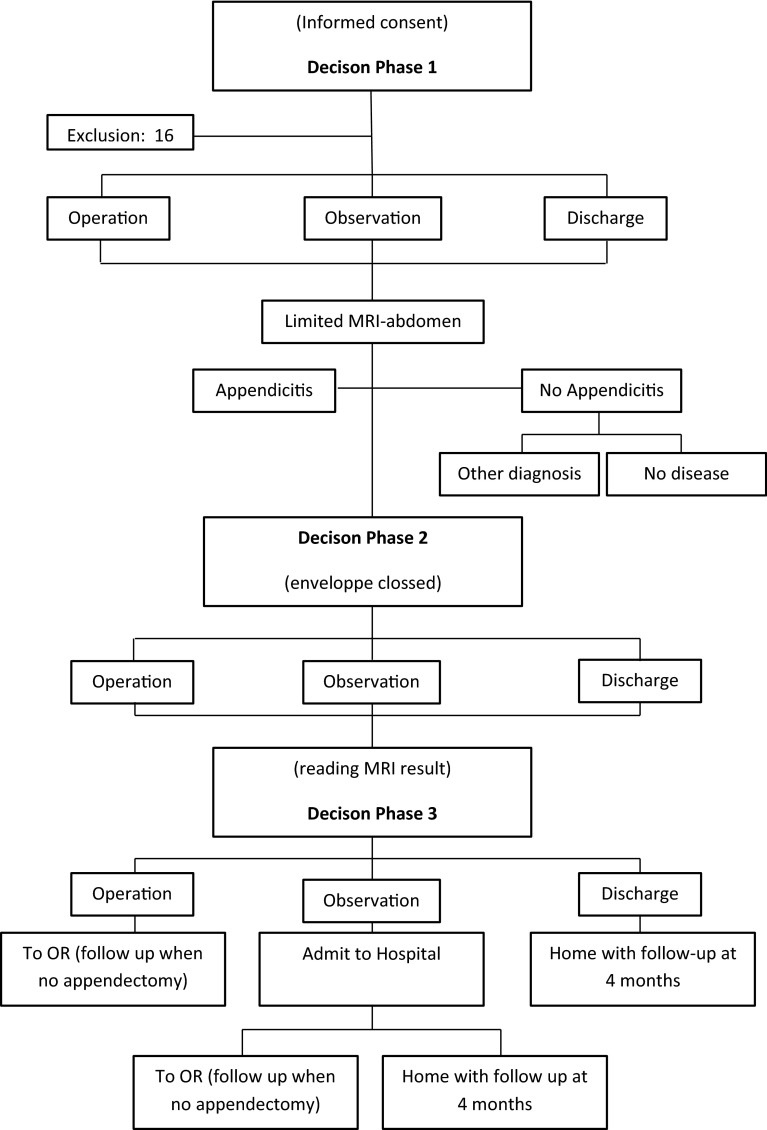
Study pathway

After clinical evaluation all included patients underwent MRI. The surgeons’ (or experienced surgical residents) diagnosis and the intended treatment, operation, observation or discharge was registered in a case record form. This was done at three decisive moments (Fig. 1).

At inclusion the patients underwent a complete routine surgical examination including patients’ history, physical examination and blood tests. After this workup all patients underwent MRI. The MRI findings were documented in a second case record form and returned to the surgeon in a sealed envelope. During the delay between the initial clinical evaluation (decision phase 1) and the moment of MRI scanning, the patient’s condition might have changed. Therefore, after the MRI the surgeon first re-evaluated the patient (decision phase 2) before reading the MRI findings. After the re-evaluation the surgeon opened the envelope, analysed the MRI findings and made his final decision (decision phase 3).

Patients designated for surgical intervention underwent laparoscopic or open appendectomy according to the surgeon’s preference. Patients selected for observation were admitted to the hospital or revised in the emergency room for re-evaluation within 24 h. Patients not having an appendectomy were followed-up by an interview by telephone 4 months after inclusion. If the patient was not treated for manifest appendicitis in that period, it was that assumed there had not been an indication for abdominal emergency surgery at the time of inclusion, and the diagnosis of acute appendicitis was considered as highly unlikely.

### MRI

In our institution we created three scanning moments for 20 min/day; at 8 a.m, 12 a.m and from 6 to 10 p.m. During our study period this worked very well. When there was no appendicitis patient to be scanned during this time, the MRI programme continued as scheduled.

All patients underwent MRI operating at a field strength of a 1.5-Tesla superconductive magnet (GyroscanIntera, Philips Medical Systems, The Netherlands). T2-weighted Turbo Spin Echo images in coronal and sagittal direction and transverse T1-weighted Gradient Echo images were obtained.

A consultant abdominal radiologist, who did not have access to the clinical findings, evaluated the results of the MRI study. The radiologist allocated the results as follows: appendicitis, other diagnosis, or no abnormality or equivocal. The MRI findings and diagnosis were written on a case record form in a sealed envelope. No other means of communication with the surgeon were allowed.

### Statistical analyses

The three clinical decisions and the MRI diagnosis were compared with the reference standard: the definitive histological diagnosis or outcome at four month’s follow-up.

Sensitivity, specificity and positive or negative predictive values of each clinical decision were calculated. To calculate a significant difference between positive predictive values and specificity at decision 1 and 3 we utilised a Chi-squared test.

## Results

In total, 128 females from the ages of 12 through to 55 receiving surgical consultation for possible acute appendicitis were seen. Sixteen out of these 128 patients were excluded from this study; nine patients underwent emergency surgery, six of whom had appendicitis and for seven other patients the MRI system was not available. Consequently, 112 patients were included (aged 12–54, median 22 years). Appendicitis was confirmed by pathology in 29/112 (26 %) patients.

**Fig. 2:**
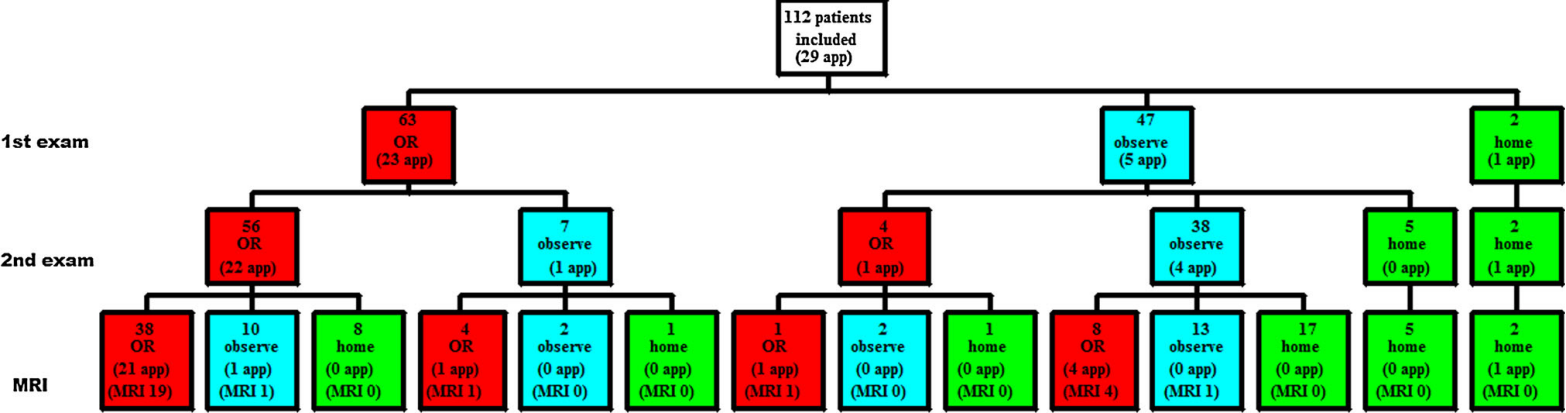
Decision-tree pathway for all included patients. At every decision the surgeon grouped the patients policy in ‘operate’ (OR), ‘observe’ or ‘discharge (home). The number of patients put in each group is depicted in the number above the policy. The number of patients who actually had appendicitis is depicted below as (…app)

The proposed treatments following the three decision phases are depicted in Fig. 2. After the initial clinical evaluation (decision 1) the proposed treatment was as follows; of the 63 patients who would have had surgery the MRI changed the decision made in 21 cases. Hence 20 patients were spared an unnecessary operation.

Of 47 patients who were admitted for observation, the MRI altered the given conclusion in 27 cases. Nine had an operation, of which five had appendicitis. The remaining four had a negative MRI but the surgeon decided to operate. 23 patients could be discharged early. Of the two patients initially designed for early discharge from the hospital, one had appendicitis on follow-up. Although the MRI result was negative, the surgeon decided to operate this patient *à froid*, because of persistent complaints. Pathology later revealed a chronic inflammation.

According to the MRI study 25 patients had appendicitis and 78 patients did not. Of these 78 patients, 22 had an alternative diagnosis (Table 1). The remaining patients had no apparent disease. In nine patients the MRI result was equivocal. One of these patients had appendicitis proven histologically at appendectomy *à froid*. MRI failed to diagnose three cases of real appendicitis (false negatives) and diagnosed no patients erroneously as having appendicitis (false positives) (Table 2).

**Table 1:** Alternate diagnoses

Diagnosis	
Ovalotoir	6
Uterus myomatosis	3
Salpingitis	3
Endometriosis cyst	2
Ovarium cyst	2
Ileitis terminalis	2
Adnexal pathology	1
Ileus	1
Bone laesion	1
Diverticulitis	1
Alternate diagnoses	22

**Table 2:** Sensitivity, specificity, positive predictive value (PPV) and negative predict value (NPV) of the MRI, based on positive or negative diagnoses only

	Definitive outcome		Sensitivity	Specificity	PPV	NPV
	Appendix +	Appendix −				
MRI						
Appendix +	25	0				
Appendix −	3	75	89 %	100 %	100 %	96 %
Equivocal	1	8				
Total	29	83				

The median time from inclusion to the end of the MRI was 1.1 h (0.4–23.2 h). The average actual visiting time (from the moment the patient arrived at the MRI until she left the unit again) was 22 min (ranging from 16 to 29 min).

The sensitivities, specificities, positive predictive values and negative predictive values for the MRI and each decision phase are depicted in Tables 2 and 3, respectively. When the MRI was conclusive, there was a positive predictive value of 100 % and negative predictive value of 96 %.

**Table 3:** Sensitivity, specificity, positive predictive value (PPV) and negative predictive value (NPV) after each decision phase

	Definitive outcome		Sensitivity	Specificity	PPV	NPV
	Appendix +	Appendix −				
Decision phase						
1						
Appendix +	28	59				
Appendix −	1	24	97 %	29 %	32 %	96 %
2						
Appendix +	27	52				
Appendix −	2	31	93 %	37 %	34 %	94 %
3						
Appendix +	28	30				
Appendix −	1	53	97 %	64 %	48 %	98 %
Total	29	83				

## Discussion

Including MRI in the third decision for appendicitis resulted in a significant decrease of surgical operative intervention and unnecessary hospitalisation without an increase in the number of missed appendicitis. The study design was unique because; firstly only females in their reproductive years were included. This specific cohort is generally considered as ‘the difficult group for diagnosing appendicitis’ [9–12]. Secondly; our gold standard was histological proven appendicitis or no signs of appendicitis at four months of follow-up. In this cohort we found a sensitivity of the MRI of 89 % and a specificity of 100 % (Table 3). The negative predictive value was 96 %. This last figure is most important because a pelvic sepsis can have serious consequences for the fertility of female patients.

Our MRI results are in accordance with earlier reports on MRI in diagnosing acute appendicitis in both male and female patients, showing good sensitivities and specificities of 97–100 and 92–97 %, respectively [13–16]. In a prospective study of 60 patients, Incesu et al. compared MRI to ultrasonography as a gold standard and reported a sensitivity of 97 % and specificity of 92 % for MRI [17].

The specificity (29 %) at decision phase one was low, but this was coupled with a high sensitivity (97 %). Besides the notoriously difficult patient cohort included in the present study, there are two other explanations for the low specificity. Firstly, as we wanted to investigate the clinical applicability in daily practice, we also allowed experienced surgical residents, instead of surgeons, to participate in the present study. Secondly, since the broad introduction of diagnostic laparoscopy at our institute, the threshold to proceed with this invasive diagnostic tool is low. At decision phase two the specificity (37 %) and sensitivity (93 %) was not significantly altered in relation to decision phase one, suggesting that only just re-examining the patient after a short amount of time was not very advantageous in improving the diagnostic accuracy. Only after including the MRI outcome in the decision tree, the specificity increased to 64 %, with a sensitivity of 97 %.

The specificity and the positive predictive value of decision 3 were lower than those of the MRI alone. This was because the actual protocol at the Department of Surgery preferred clinical evaluation over the new imaging techniques. So, despite a negative MRI result, the surgeon could decide whether or not to have the patient undergo surgical intervention. This occurred in 26 patients with a negative MRI result. Two of them had appendicitis. Therefore our study shows a high number of negative appendectomies (47 %) in this selected group. This was previously reported to be of between 35 and 45 % [2, 3]. We believe that if the clinicians become confident with the MRI assessment the number of negative appendectomies will decrease without an increase in missed appendicitis. In the study of Cobben et al., they reported a very low negative appendectomy rate of 3 %, where they combined the MRI and sonographic result [14]. They also observed the effect of an MRI scan of the appendix on the use of hospital resources and concluded that an abdominal MRI in the evaluation of patients suspected of having appendicitis is a reliable, safe and potentially cost-effective technique. In contrast with our study they included all patients, men/women and the young/old, with possible appendicitis. Moreover, their imaging technique was slightly different, with a breath hold MRI following scout images instead of a limited MRI of the lower right abdomen. We did not perform any diffusion weighted imaging (DWI), although in a recent publication DWI showed to be a promising diagnostic tool in showing disturbed diffusion at the site of the inflamed appendix [18].

Of the three patients with false negative MRI results, two were operated on directly due to the severe clinical symptoms of peritonitis. One patient was operated *à froid* 2 months after the MRI because of consistent complaints. As all other patients recovered well during their follow-up, we assume that there were no further false negative MRI results, and that a self-limiting disease was the cause of the initial symptoms. Therefore, if an entity such as endoappendicitis, which was not recognisable with MRI, exists, this was of no clinical importance to our study.

In nine patients the MRI result was equivocal. In all of these cases appendicitis could not be excluded completely. In four cases the appendix was not detectable, but there were no secondary signs of appendicitis. In two cases the terminal ileum was also enlarged, therefore correctly termed as terminal ileitis. In one case there was doubt if the enlarged structure was the appendix or an infected Meckel's diverticula, however this appeared to be a Meckel's diverticulitis. In one other case the enlarged appendix was found next to an enlarged adnex that had signs of infection. This showed the manifestation of adnexitis. In the last case the appendix diameter was slightly enlarged to a maximum of 8 mm but there were no secondary signs of appendicitis. On pathology there were no symptoms of infection (Table 4). We didn’t calculate the positive or negative predictive values with the equivocal results.

**Table 4:** The nine equivocal MRI results

Appendix not visible, no secondary signs of appendicitis	4
Appendix enlarged, but no secondary signs of appendicitis	1
Enlarged terminal ileum, together with enlarged appendix	2
Enlarged adnex, together with enlarged appendix	1
Meckel's diverticula or enlarged appendix	1
Total	9

None of these patients had appendicitis on OR or follow-up

Several other diagnostic tools like ultrasound, CT scan and diagnostic laparoscopy were used in daily practice to confirm or exclude the diagnosis of appendicitis. In the Dutch guidelines about the diagnosis of appendicitis, which was published in 2010, it advises for an ultrasound in order to minimise the negative appendectomy ratio. And in case of a negative or inconclusive ultrasound the guidelines prescribe to perform a CT scan. The use of a CT scan is known to have a good sensitivity (87–100 %) and specificity (83–98 %) [19]. However, especially in this group of young females, the use of ionising radiation must be reduced as much as possible. In experienced hands ultrasound can be an attractive alternative with a large sensitivity ranging from 75 to 96 % and specificity of 85 % to near 100 %, however the technique is known to be very operator dependent [7, 19, 20].

Diagnostic laparoscopy is found to be of great use in diagnosing appendicitis especially in females [10, 11]. Van den Broek et al. found that diagnostic laparoscopy reduced the negative appendectomy rate in women from 39 to 20 % [10]. Nevertheless the disadvantages of diagnostic laparoscopy, the use of general anaesthesia, the morbidity that accompanies an invasive examination and the hospitalisation including costs of operation room and equipment all together make the diagnostic laparoscopy an expensive diagnostic method. Using MRI as a non-invasive tool such disadvantages could be prevented.

In the present study nine patients presented with clear severe peritonitis were excluded for MRI, in all other patients the short time between admission and MRI was not detrimental to any.

Despite the advantages of MRI, such as not using ionising radiation and the ability to give reproducible images, when compared with CT, MRI is said to not be easily accessible for emergency studies. In the research period of 1.5 years we had time slots on the MRI, only seven patients were excluded in this study since the MRI was not available.

In conclusion, in young females clinical diagnosis of appendicitis is notoriously difficult [11]. Imaging tools are needed to determine the correct diagnosis with a minimum of fertile invasive procedures. Our study shows that the application of MRI in this particular patient group of fertile females improves the clinical decision-making process by reducing the surgical intervention rate and moving patients early to the appropriate treatment group.
